# Primary Site and Other Prognostic Factors for Fibrosarcoma: An Analysis of the National Cancer Database

**DOI:** 10.7759/cureus.19163

**Published:** 2021-10-31

**Authors:** Mary Dahl, Sarah J Aurit, Peter T Silberstein, Jonathan Gootee

**Affiliations:** 1 Department of Internal Medicine, Division of Hematology/Oncology, Creighton University School of Medicine, Omaha, USA; 2 Division of Clinical Research and Evaluative Sciences, Creighton University School of Medicine, Omaha, USA; 3 Department of Surgery, West Virginia University School of Medicine, Morgantown, USA

**Keywords:** ncdb, prognostic factors, insurance, socioeconomic, primary anatomical site, fibrosarcoma

## Abstract

Purpose: Fibrosarcoma (FS) is a rare and malignant tumor that can occur in a variety of anatomic sites. The goal of this study is to use the National Cancer Database (NCDB) to analyze various factors affecting overall survival in FS and to be one of the rare studies to characterize the significance of the primary anatomic sites.

Methods: The study cohort included 2,278 patients diagnosed with fibrosarcoma who received surgery from the NCDB. Kaplan-Meier curves, log-rank tests, and a multivariable Cox proportional hazard model were used to analyze the significance of factors affecting overall survival.

Results: The head, face, and neck (HR = 1.44; 95% CI: 1.01-2.05; *P* = 0.046) and thorax anatomical sites (HR = 1.33; 95% CI: 1.02-1.73; *P* = 0.035) had a higher increased risk of death in comparison to the lower limb and hip. Compared to patients with private insurance, patients without insurance (HR = 1.99; 95% CI: 1.22 to 3.25; P = 0.006) and patients with Medicaid (HR = 1.99; 95% CI: 1.37 to 2.90; P < 0.001) had decreased overall survival. Patients associated with a zip code-level median household income ≥ $63,000 had a decreased risk of mortality when compared to lower income groups.

Conclusion: In general, older patients with comorbidities, advanced-stage disease, and larger tumors who did not have private insurance and were from areas associated with lower income levels had poorer overall survival. No significant difference in overall survival was associated with receipt of neoadjuvant chemotherapy or neoadjuvant radiation.

## Introduction

Fibrosarcoma (FS) is a rare mesenchymal tumor. The World Health Organization classifies adult FS as “a malignant tumor, composed of fibroblasts with variable collagen production and, in classical cases, a herringbone architecture” [1]. FS is distinct from other sarcoma subtypes of spindle-shaped cell origin and is diagnosed as a diagnosis of exclusion. While FS was once thought to be a common soft tissue tumor, its incidence has been decreasing as the classification of true FS has become more well-defined. Surveillance, Epidemiology, and End Results (SEER) data suggest FS represents approximately 3.6% of all adult soft tissue sarcomas [2]; however, a recent study estimates the true incidence of FS to represent less than 1% of all adult soft tissue sarcomas [3]. FS occurs in a wide variety of anatomic locations including the extremities, head and neck, heart, gastrointestinal (GI) tract, kidney, and reproductive tract [4-9]. Age at diagnosis varies greatly with a median of around 50 years of age [3].

The standard treatment for fibrosarcoma is surgical excision with neoadjuvant or adjuvant radiation therapy in some cases. Chemotherapy is sometimes discussed, although the effectiveness of this has not been conclusive [10,11].

FS is malignant with a previously reported overall five-year survival rate between 40% and 60%, regardless of grade [12]. Understanding factors that affect prognosis is important for patients and clinicians. However, due to the rarity of cases, factors affecting the overall survival of FS have not been comprehensively studied. Male sex, older age, advanced stage, higher grade, increased tumor size, and not receiving surgical treatment have been reported to be associated with decreased overall survival [13]. The goal of our study is to further assess prognostic variables for FS. The primary anatomic site has not previously been examined, and this paper plans on elucidating the impact it has on survival. We examine the association between overall survival and age, sex, race, income, education, insurance status, tumor size, analytical stage, neoadjuvant chemotherapy, neoadjuvant radiation, distance traveled for health care, and Charlson-Deyo comorbidity score. Our aim is to be the largest study to assess prognostic factors for fibrosarcoma.

This article was previously presented as a poster at the American Society of Clinical Oncology Medical Student & Resident Abstract Forum on June 10th, 2021.

## Materials and methods

This is a retrospective cohort study of patients diagnosed with fibrosarcoma from 2004 to 2016. Patients were identified from the National Cancer Database (NCDB). NCDB is sponsored by the American College of Surgeons and the American Cancer Society. It collects data from more than 1,500 Commission on Cancer-accredited facilities and includes over 70% of new cancer diagnoses in the United States. Authors were granted access to de-identified patient data from the NCDB through the Participant User Data Files program.

Patients with FS were identified from NCDB data using ICD-O-3 codes 8810, 8811, 8812, and 8813. Patients were excluded from the cohort if they had concurrent tumors, were associated with a primary anatomic site with less than 100 cases, or had any missing clinical or demographic factors. Patients who did not receive surgical treatment were also excluded. Since surgical excision is the standard treatment for FS, this excluded a small number of patients who did not receive the standard treatment. The final cohort used for the study was comprised of 2,278 patients.

Patients were analyzed by age, sex, race, income, education, insurance status, tumor size, analytical stage, primary anatomic site, adjuvant and neoadjuvant chemotherapy and radiation, distance traveled for health care, and Charlson-Deyo comorbidity score. The race was categorized into three groups: White, African-American, and Other. The race group categorized as other includes American Indian, Aleutian or Eskimo, Chinese, Japanese, Filipino, Hawaiian, Korean, Vietnamese, Kampuchean, Asian Indian or Pakistani NOS, Asian Indian, Micronesian NOS, Other Asian, Asian NOS, Oriental NOS, and Pacific Islander NOS. Income was measured by median household income from 2008 to 2012 for the zip code where the patient resided at the time of diagnosis. Education was measured by the percentage of residents within the patient’s zip code of residence who did not graduate from high school between 2008 and 2012. The staging was measured by NCDB analytical stage. The variable NCDB analytical stage includes the pathologic staging when available; if not available, then clinical staging is utilized. Insurance status was categorized into five groups: uninsured, private, Medicare, Medicaid, and other government insurance. The primary anatomical site was divided into seven groups using ICD-O-3 topography codes: head or face or neck, lower limb or hip, thorax, abdomen, pelvis, trunk, and other soft tissues. Distance traveled for health care was measured by the miles between the patient’s residence and the hospital that reported the case. Comorbidities were measured by Charlson-Deyo score, and the patients were divided into groups with scores of 0, 1, and ≥2.

Kaplan-Meier curves were plotted, and the survival tables were used to calculate median overall survival at five and 10 years for the variables of interest. A multivariable Cox hazard regression model was used to identify independent prognostic factors. Variables included in the multivariable Cox model were age, sex, race, income, education, insurance status, tumor size, analytical stage, primary anatomic site, neoadjuvant chemotherapy, neoadjuvant radiation, distance traveled for health care, and Charlson-Deyo comorbidity score. For the multivariable Cox model, five-year increments were used for age, 10-mm increments were used for tumor size, and 50-mile increments were used for distance traveled for health care. We accounted for the correlation of patients within the same facility with a robust sandwich covariance matrix. The functional form of continuous variables was examined with loess methods; the proportional hazards assumption for each variable was evaluated with log-negative-log survival curves and statistical interaction with time.

Descriptive statistics and unadjusted survival analysis were conducted using IBM Statistical Package for the Social Sciences (SPSS) version 27 (IBM Corp., Armonk, NY). The multivariable survival analysis was conducted with SAS version 9.4 (SAS Institute Inc., Cary, NC); P < 0.05 indicated statistical significance.

## Results

Patient characteristics

Descriptive statistics for the cohort are displayed in Tables 1-3. The cohort had a slight male predominance (52.2%), and the overwhelming majority was white (86.7%). The median age at diagnosis was 59 years of age. Patients were spread across the four income brackets with the highest percentage in the highest income group (37.3%). Similarly, patients were spread across the four education level groups with the highest percentage in the second most educated group (32.3%). The majority of patients had private insurance (53.7%), followed by Medicare (35.3%). Patients were relatively healthy with 82.4% having a Charlson-Deyo score of 0. The median tumor size at diagnosis was 6.5 cm. Patients generally had earlier-stage disease with 72% having an NCDB analytical stage of I or II. The lower limb or hip was the most common tumor site encompassing 62.5% of the cohort, followed by the thorax with 9.7%. The most common histology was fibromyxosarcoma (78.8%), followed by fibrosarcoma NOS (21.1%). Only three patients had periosteal fibrosarcoma or fascial fibrosarcoma (0.1%). All patients in the cohort received surgical treatment with a minority of patients also receiving neoadjuvant or adjuvant therapy. Adjuvant radiation was the most common additional treatment (36.0%), followed by neoadjuvant radiation (14.4%). Chemotherapy was less common with 4.3% of the cohort receiving adjuvant chemotherapy and 4.0% receiving neoadjuvant chemotherapy.

**Table 1:** Clinical and demographic characteristics of 2,278 patients with fibrosarcoma

Variable	N = 2,278	% of total
Sex		
Male	1190	52.2
Female	1088	47.8
Race		
White	1974	86.7
African-American	225	9.9
Other	79	3.5
Age (years)		
Mean ± Standard deviation	57.44 ± 18.167	
Median (interquartile range)	59.00 (46.00-71.00)	
Zip code-level median household income (2008-2012, $)		
< $38,000	345	15.1
$38,000-$47,999	510	22.4
$48,000-$62,999	574	25.2
≥ $63,000	849	37.3
Zip code-level education (% without high-school degree, 2008-2012)		
≥ 21%	357	15.7
13%-20.9%	494	21.7
7%-12.9%	735	32.3
< 7%	692	30.4
Insurance status		
Uninsured	81	3.6
Private	1224	53.7
Medicaid	122	5.4
Medicare	804	35.3
Other government	47	2.1
Distance traveled for health care (miles)		
Mean ± Standard deviation	51.64 ± 142.980	
Median (interquartile range)	16.10 (6.60-50.33)	
Charlson-Deyo comorbidity score		
0	1878	82.4
1	310	13.6
≥ 2	90	4.0

**Table 2:** Tumor characteristics of 2,278 patients with fibrosarcoma NCDB, National Cancer Database.

Variable	N = 2,278	% of total
Primary anatomic site		
Head, face, neck	133	5.8
Lower limb or hip	1424	62.5
Thorax	222	9.7
Abdomen	114	5
Pelvis	206	9
Trunk	151	6.6
Other	28	1.2
Tumor size (mm)		
Mean ± Standard deviation	97.76 ± 131.127	
Median (interquartile range)	65.00 (40.00-110.00)	
NCDB analytical stage		
1	970	42.6
2	669	29.4
3	575	25.2
4	64	2.8
Histology		
Fibrosarcoma, NOS	481	21.1
Fibromyxosarcoma	1794	78.8
Periosteal fibrosarcoma	1	<0.1
Fascial fibrosarcoma	2	0.1

**Table 3:** Treatment characteristics of 2,278 patients with fibrosarcoma

Variable	N = 2,278	% of total
Adjuvant therapy		
Received adjuvant chemotherapy	97	4.3
Received adjuvant radiation	821	36.0
Neoadjuvant therapy		
Received neoadjuvant chemotherapy	91	4.0
Received neoadjuvant radiation	328	14.4
Surgical margins		
No residual tumor	1861	81.7
Residual tumor, NOS	162	7.1
Microscopic residual tumor	237	10.4
Macroscopic residual tumor	18	0.8

Survival analysis

Overall survival for the cohort is displayed in Figure 1. Survival by variables is displayed in Figures 2-8. Five- and 10-year survival rates were found to be 75.0% and 52.6%, respectively. Table 4 displays five- and 10-year survival estimates by variable. Multivariable Cox model results are found in Table 5.

**Figure 1:**
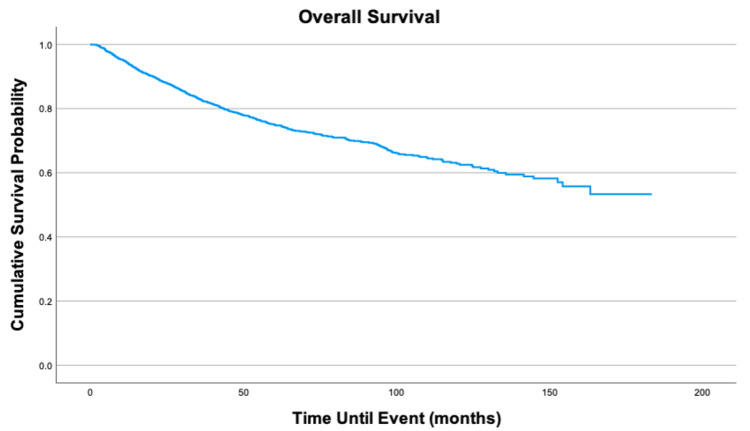
Overall survival of 2,278 patients with fibrosarcoma

**Figure 2:**
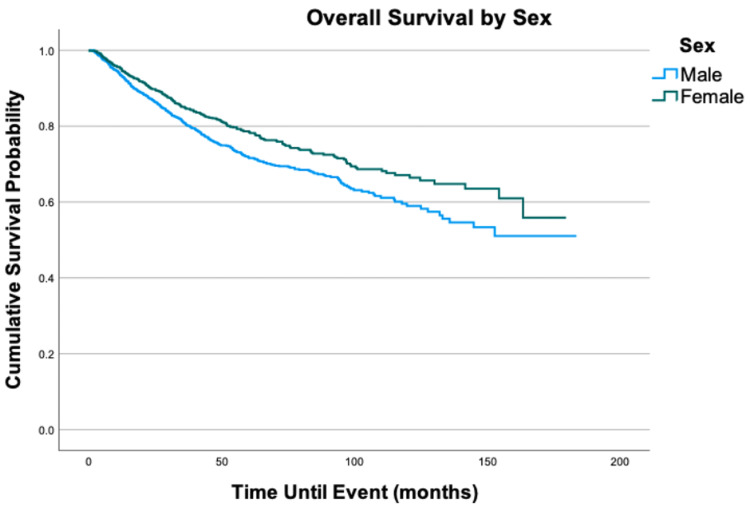
Overall survival by sex of 2,278 patients with fibrosarcoma, P = 0.001

**Figure 3:**
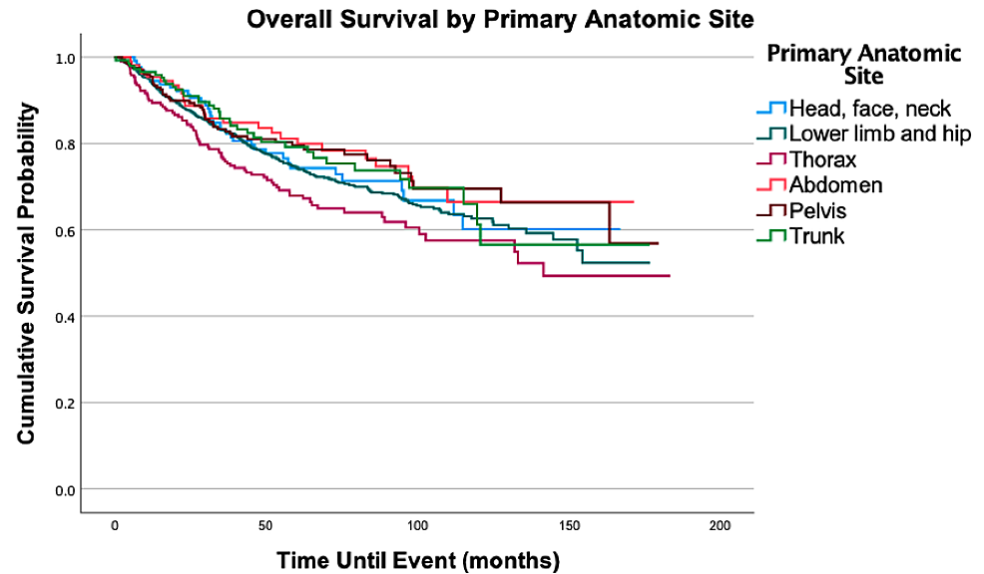
Overall survival by the primary anatomic site of 2,278 patients with fibrosarcoma, P = 0.057

**Figure 4:**
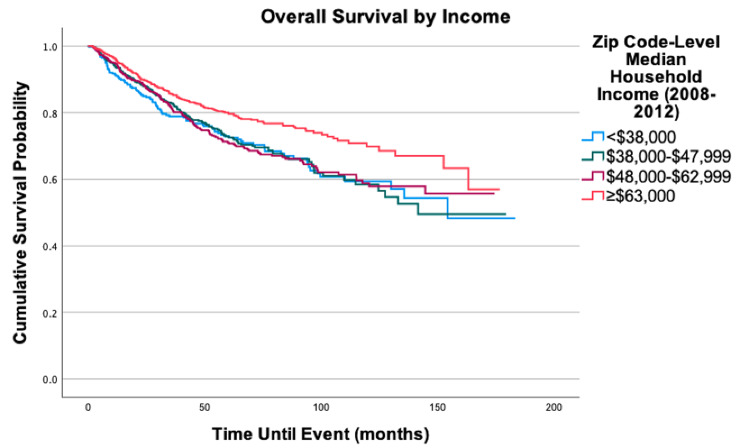
Overall survival by income of 2,278 patients with fibrosarcoma, P = 0.001

**Figure 5:**
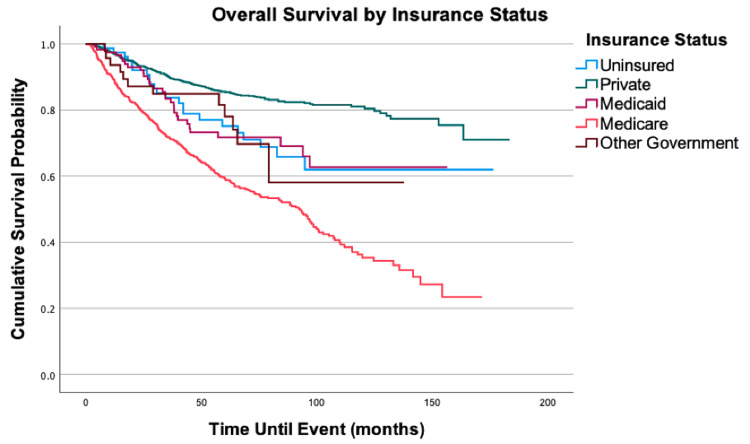
Overall survival by insurance status of 2,278 patients with fibrosarcoma, P < 0.0001

**Figure 6:**
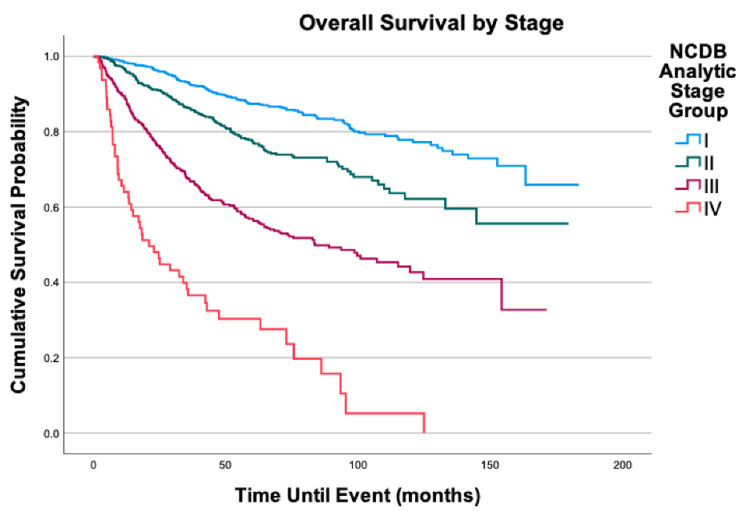
Overall survival by stage of 2,278 patients with fibrosarcoma, P < 0.0001

**Figure 7:**
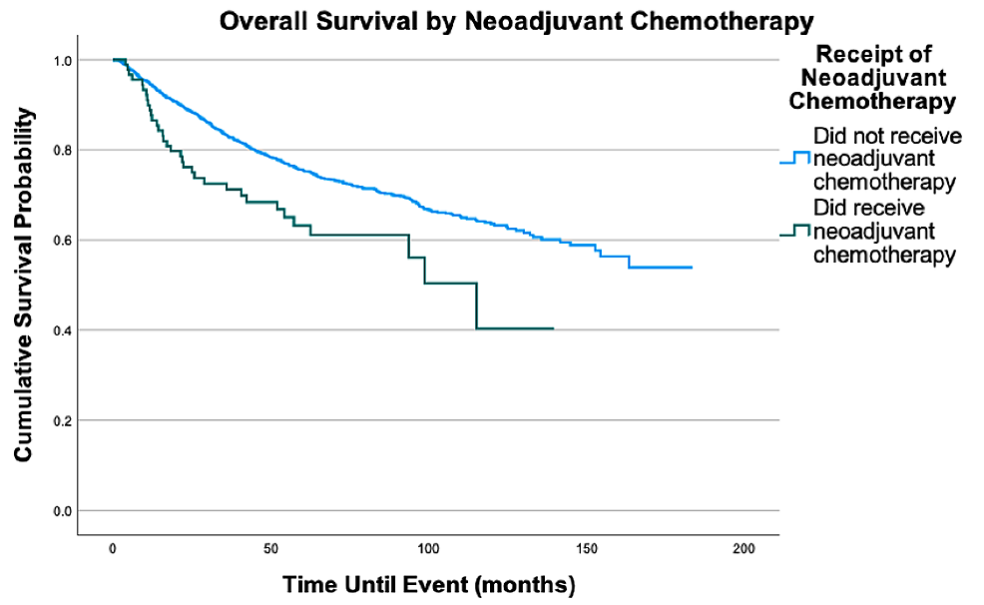
Overall survival by neoadjuvant chemotherapy of 2,278 patients with fibrosarcoma, P = 0.003

**Figure 8:**
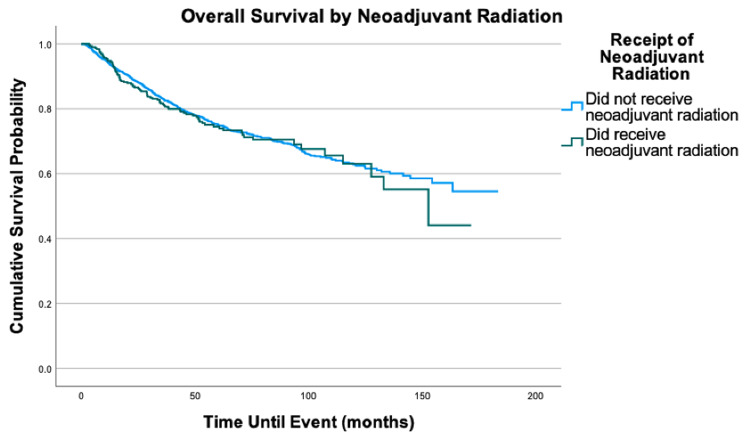
Overall survival by neoadjuvant radiation of 2,278 patients with fibrosarcoma, P = 0.699

**Table 4:** Median five- and 10-year survival estimates of 2,278 patients with fibrosarcoma * Information unavailable.

Variable	5-year (%)	10-year (%)
Overall	75.0	62.5
Sex		
Male	71.7	59.0
Female	78.5	66.4
Race		
White	74.4	62.0
African-American	76.5	62.9
Other	85.5	*
Zip code-level median household income (2008-2012, $)		
< $38,000	72.5	57.1
$38,000-$47,999	72.6	56.6
$48,000-$62,999	70.8	57.9
≥ $63,000	79.8	69.8
Zip code-level education (% without high-school degree, 2008-2012)		
≥ 21%	73.7	63.4
13%-20.9%	72.8	56.7
7%-12.9%	74.0	59.2
< 7%	77.9	68.9
Primary anatomic site		
Head, face, neck	74.4	60.2
Lower limb and hip	74.5	62.7
Thorax	67.3	54.9
Abdomen	80.0	66.6
Pelvis	78.7	66.4
Trunk	78.0	56.6
Other	85.7	*
Age (years)		
0-25	*	*
26-50	88.5	83.3
51-75	74.1	59.7
76-100	50.1	18.7
NCDB analytical stage		
I	87.2	77.3
II	77.3	62.2
III	56.6	42.7
IV	27.6	*

**Table 5:** Multivariable Cox regression model of 2,278 patients with fibrosarcoma

Variable	HR (95% Confidence Interval)	P values
Age (5 years)	1.52 (1.37-1.67)	<0.001
Great circle distance (50 miles)	1.00 (0.99-1.00)	0.831
Males vs. females	1.17 (0.99-1.39)	0.058
Race and ethnicity		
African-American vs. White	1.19 (0.89-1.60)	0.247
Other vs. White	0.61 (0.36-1.01)	0.056
African-American vs. Other	1.96 (1.09-3.53)	0.025
Charlson-Deyo Score		
1 vs. 0	1.30 (1.03-1.64)	0.029
≥ 2 vs. 0	1.69 (1.22-2.35)	0.002
1 vs. ≥ 2	0.77 (0.53-1.10)	0.152
Zip code-level median household income (2012 US Dollars)		
< 48,000-62,999 vs. ≥ 63,000	1.29 (1.03-1.62)	0.027
< 38,000-47,999 vs. ≥ 63,000	1.35 (>1.00-1.81)	0.048
< 38,000 vs. ≥ 63,000	1.51 (1.03-2.20)	0.033
< 38,000-47,999 vs. < 48,000-62,999	1.04 (0.80-1.36)	0.762
< 38,000 vs. < 48,000-62,999	1.17 (0.82-1.66)	0.386
< 38,000 vs. < 38,000-47,999	1.12 (0.82-1.53)	0.466
Zip code-level education (2008-2012, % No high-school diploma)		
7-12.9 vs. < 7	1.11 (0.90-1.38)	0.331
13-20.9 vs. < 7	1.02 (0.77-1.35)	0.904
≥ 21 vs. < 7	0.90 (0.61-1.32)	0.585
13-20.9 vs. 7-12.9	0.92 (0.74-1.14)	0.427
≥ 21 vs. 7-12.9	0.81 (0.59-1.11)	0.195
≥ 21 vs. 13-20.9	0.88 (0.65-1.19)	0.418
Insurance		
None vs. Private	1.99 (1.22-3.25)	0.006
Medicaid vs. Private	1.99 (1.37-2.90)	<0.001
Medicare vs. Private	1.21 (0.91-1.60)	0.191
Other government vs. Private	1.27 (0.75-2.14)	0.377
None vs. Other government	1.57 (0.74-3.33)	0.239
Medicaid vs. Other government	1.57 (0.84-2.94)	0.156
Medicare vs. Other government	0.95 (0.54-1.68)	0.864
None vs. Medicare	1.65 (0.95-2.85)	0.074
Medicaid vs. Medicare	1.65 (1.12-2.43)	0.011
None vs. Medicaid	1.00 (0.55-1.82)	0.997
Primary site		
Head, face, neck vs. lower limb and hip	1.44 (1.01-2.05)	0.046
Thorax vs. lower limb and hip	1.33 (1.02-1.73)	0.035
Abdomen vs. lower limb and hip	1.08 (0.73-1.60)	0.709
Pelvis vs. lower limb and hip	0.99 (0.71-1.39)	0.959
Trunk vs. lower limb and hip	0.98 (0.70-1.37)	0.887
Other vs. lower limb and hip	0.33 (0.11-0.98)	0.045
Head, face, neck vs. other	4.33 (1.38-13.60)	0.012
Thorax vs. other	4.00 (1.33-12.04)	0.014
Abdomen vs. other	3.25 (1.04-10.12)	0.042
Pelvis vs. other	2.99 (0.97-9.21)	0.057
Trunk vs. other	2.94 (0.94-9.17)	0.063
Head, face, neck vs. trunk	1.47 (0.92-2.35)	0.106
Thorax vs. trunk	1.36 (0.90-2.05)	0.140
Abdomen vs. trunk	1.10 (0.67-1.82)	0.697
Pelvis vs. trunk	1.02 (0.63-1.63)	0.947
Head, face, neck vs. pelvis	1.45 (0.91-2.30)	0.117
Thorax vs. pelvis	1.34 (0.89-2.02)	0.164
Abdomen vs. pelvis	1.09 (0.67-1.77)	0.736
Head, face, neck vs. abdomen	1.33 (0.79-2.24)	0.281
Thorax vs. abdomen	1.23 (0.79-1.93)	0.361
Head, face, neck vs. thorax	1.08 (0.70-1.66)	0.721
Tumor Size (10 mm)	1.0007 (1.0002-1.0012)	0.009
Neoadjuvant chemotherapy vs. no neoadjuvant chemotherapy	1.19 (0.69-2.05)	0.534
Neoadjuvant radiation vs. no neoadjuvant radiation	0.91 (0.69-1.19)	0.495
Analytic stage		
Stage II vs. Stage I	1.30 (1.05-1.63)	0.018
Stage III vs. Stage I	2.53 (2.02-3.16)	<0.001
Stage IV vs. Stage I	7.79 (5.16-11.78)	<0.001
Stage III vs. Stage II	1.94 (1.61-2.34)	<0.001
Stage IV vs. Stage II	5.97 (4.06-8.78)	<0.001
Stage IV vs. Stage III	3.08 (2.11-4.50)	<0.001

Older age was associated with an increased risk of death. Five- and 10-year survival estimates decreased as age increased, with the oldest age group of 76-100 years of age having the lowest survival rates at 50.1% and 18.7%, respectively. Upon multivariable analysis, an increase in five years of age was associated with a 52% increase in mortality (HR = 1.52; 95% CI: 1.37-1.67, P < 0.001). With respect to sex, females had a slightly higher unadjusted survival at both five and 10 years (78.5% and 66.4%) compared to males (71.7% and 59.0%). However, after adjusting for other variables in the multivariable Cox model, no difference was found in the overall survival between males and females (HR = 1.17; 95% CI: 0.99-1.39, P = 0.058). Patients whose race was categorized as other had the best five-year survival at 85.5%, compared to 76.5% for African Americans and 74.4% for whites. After adjusting for other variables, African Americans had a 96% increase in mortality when compared to patients of other races (HR = 1.96; 95% CI: 1.09-3.53, P = 0.025). When compared to patients with a Charlson-Deyo score of 0, patients with a score of 1 and ≥2 had a 30% (HR = 1.30; 95% CI: 1.03-1.64, P = 0.029) and 69% (HR = 1.69; 95% CI: 1.22-2.35, P = 0.002) increase in mortality risk, respectively.

There was an increase in mortality when comparing all three lower levels of income to the highest income bracket of ≥$63,000: <$38,000 (HR = 1.51, 95% CI: 1.03-2.20, P = 0.033), $38,000-$47,999 (HR = 1.35; 95% CI: >1.00-1.81, P = 0.048), $48,000-$62,999 (HR = 1.29; 95% CI: 1.03-1.62, P = 0.027). Compared to patients with private insurance, patients without insurance (HR = 1.99, 95% CI: 1.22-3.25, P = 0.006) and patients with Medicaid (HR = 1.99, 95% CI: 1.37-2.90, P < 0.001) had decreased the overall survival. Patients on Medicaid also had a 65% increased mortality risk (HR = 1.65, 95% CI: 1.12-2.43, P = 0.011) when compared to patients on Medicare.

Regarding primary anatomic site, after adjusting for all else, the head, face, and neck (HR = 1.44; 95% CI: 1.01-2.05, P = 0.046) and thorax (HR = 1.33; 95% CI: 1.02-1.73, P = 0.035) had increased mortality when compared to the lower limb and hip. Five- and 10-year survival probabilities steadily decreased as stage increased. Stage II had a 30% increased mortality risk when compared to stage I (HR = 1.30, 95% CI: 1.05-1.63, P = 0.018); Stage III was also associated with increased mortality risk (HR = 2.53, 95% CI: 2.02-3.16, P < 0.001). Finally, Stage IV had even greater increased mortality risk in a similar comparison (HR = 7.79; 95% CI: 5.16-11.78, P < 0.001).

No significant difference in the overall survival was associated with the distance traveled to health care, education level, neoadjuvant chemotherapy, or neoadjuvant radiation. If a patient received neoadjuvant chemotherapy, our data showed they did not receive adjuvant chemotherapy. Thus, the inclusion of neoadjuvant chemotherapy in the multivariable model fully explains both neoadjuvant and adjuvant chemotherapy. The same pattern with neoadjuvant and adjuvant therapy was found for radiation.

## Discussion

Previous research on fibrosarcoma has been limited due to its low prevalence. This is one of the largest studies examining prognostic factors for fibrosarcoma and a rare one to characterize the significance of the primary anatomic site.

Many of the descriptive statistics for our patient cohort provide support for the previously reported characteristics of patients diagnosed with FS. We found that FS is slightly more common in males than females, which is consistent with other sources that report a very slight male predominance [3,12,13], although an equal sex incidence has also been reported [1,13]. If there is a true difference in sex incidence, it is likely minimal.

Our study is consistent with the current understanding that the most common primary anatomical site is the lower extremities [3,12]. This is encouraging as we found a significant decrease in mortality risk for tumors in the lower limb and hip when compared to the head, face, and neck or the thorax.

The median tumor size at diagnosis was 6.5 cm in both our study as well as a study of 163 patients at the Mayo Clinic [3]. While increased tumor size is correlated with a worse prognosis, tumor size has a smaller impact when compared to other factors such as primary anatomical site and stage. NCDB's analytical stage is reflective of the degree of metastasis and progression of cancer. While our analysis cannot distinguish between fibrosarcoma-specific mortality and all-cause mortality, the strong association between stage and mortality suggests these patients died of cancer. As expected, this emphasizes the importance of early detection and diagnosis of fibrosarcoma as patients diagnosed at an earlier stage and with smaller tumors have better survival outcomes.

Surgery is currently the standard treatment for fibrosarcoma [11]. Radiation therapy is not standard but can be recommended due to a variety of factors, which include tumors that are high grade and deep [10,11]. This is reflected in our cohort as adjuvant radiation therapy was the most common additional treatment modality with 36.0% of the cohort receiving adjuvant radiation and 14.4% receiving neoadjuvant radiation. Chemotherapy, however, has no clearly defined guidelines for utility in fibrosarcoma patients [11]. Fibrosarcoma cells have been found to be associated with resistance to multiple chemotherapeutic agents that may help explain its potential lack of benefit [14]. Thus, chemotherapy is less common, which is reflected in our cohort as only 4.3% received adjuvant chemotherapy and 4.0% received neoadjuvant chemotherapy. It has been suggested that neoadjuvant chemotherapy may provide more benefit than adjuvant chemotherapy in large, high-grade soft tissue sarcomas [15]. However, our multivariable analysis reveals no significant mortality difference based on receipt of neoadjuvant chemotherapy. Similarly, no mortality benefit was found associated with neoadjuvant radiation. While patients receiving neoadjuvant chemotherapy or neoadjuvant radiation were more likely to have the higher-stage disease, the lack of mortality difference suggests there may not be any survival benefit to these neoadjuvant treatment options. Randomized controlled trials would need to be performed to evaluate the effectiveness of neoadjuvant treatments in FS more definitively; however, our results fail to provide support for any mortality benefit associated with neoadjuvant chemotherapy or radiation.

Socioeconomic factors have been found to be significant prognostic factors in other sarcomas [16,17], but their specific impact in FS is yet to be analyzed. Our multivariable Cox model revealed a 99% increase in mortality risk for patients with Medicaid or no insurance when compared to patients with private insurance. Additionally, patients in the highest income bracket had a statistically significant survival benefit when compared to patients in all lower-income brackets. The reasons explaining these mortality differences are likely multifactorial. Recent SEER-based studies have suggested that an association exists between insurance status and the presence of metastases at the diagnosis for certain soft tissue sarcoma subtypes [18,19]. Additionally, an NCDB study found that Medicare, Medicaid, or no insurance was correlated with increased time to treatment initiation, whereas median income > $63,000 was associated with shorter time to treatment initiation [20]. While the further analysis did not reveal these associations in our study cohort, there was an association between facility type and insurance status (P = 0.031). A larger proportion of patients with Medicaid or no insurance was treated at a community cancer center (9.3% and 6.3%, respectively), compared to patients with Medicare or private insurance (4.2% and 3.2%, respectively). Facility type has been noted to be an independent factor affecting prognosis in other cancers [21-23], with community cancer centers usually having poorer overall survival when compared to academic/research programs, comprehensive community cancer programs, or integrated network cancer programs. Furthermore, facility volume is often correlated with facility type. An NCDB study on soft tissue sarcoma of the extremities found that treatment at higher volume centers compared to lower volume centers may be associated with decreased mortality risk [24]. Facility volume is reflected in the Commission on Cancer facility category assignments with community cancer programs seeing between 100 and 500 newly diagnosed cases of cancer per year and comprehensive community cancer programs as well as academic programs seeing more than 500 per year. Further studies may be able to elucidate reasons for the associations between insurance status, facility type, facility volume, and overall survival. This could potentially lead to improved referral of patients to certain facilities or interventions to improve the accessibility of such facilities to all patients.

Our five-year overall survival rate of 75% is higher than the <55% value reported from a study of 26 patients with FS [3] and the previously published rate of 40%-60% [12]. One factor in this could be the selection of only surgical candidates for our study as surgery is the standard treatment and has been shown to be associated with a more favorable prognosis [13].

One significant strength of our study is the relatively large sample size, given the rarity of fibrosarcoma. NCDB includes over 70% of new cancer diagnoses in the United States and is considered a comprehensive database. However, it does not include data from patients diagnosed at non-Commission on Cancer (CoC)-accredited facilities. Thus, we do not know if non-CoC patient data would be consistent with CoC patient data found on NCDB. Another limitation of our conclusions is the retrospective nature of the study. We were only able to use variables gathered by NCDB, and our cohort was limited due to patients with missing data. Lastly, NCDB only records overall survival and does not record cancer-specific survival. Thus, we are unable to differentiate between fibrosarcoma-specific mortality and all-cause mortality. However, the consistency between many of our results and those published in current literature is encouraging and provides support to our conclusions.

## Conclusions

We identified various factors associated with increased mortality in fibrosarcoma patients. Tumors located in the head, face, and neck or tumors in the thorax were associated with increased mortality when compared to tumors in the lower limb and hip. In general, older patients with comorbidities, advantaged stage disease, and larger tumors who did not have private insurance and were from areas associated with lower income levels had poorer overall survival. Additionally, no survival benefit was found with neoadjuvant chemotherapy or neoadjuvant radiation. These conclusions are helpful for clinical knowledge of fibrosarcoma as the understanding of this rare tumor continues to become more well-defined.
